# Inclusion of a Furin Cleavage Site Enhances Antitumor Efficacy against Colorectal Cancer Cells of Ribotoxin α-Sarcin- or RNase T1-Based Immunotoxins

**DOI:** 10.3390/toxins11100593

**Published:** 2019-10-12

**Authors:** Javier Ruiz-de-la-Herrán, Jaime Tomé-Amat, Rodrigo Lázaro-Gorines, José G. Gavilanes, Javier Lacadena

**Affiliations:** 1 Departamento de Bioquímica y Biología Molecular, Facultad de Ciencias Químicas, Universidad Complutense de Madrid, Madrid 28040, Spain; chaosrorri@hotmail.com (J.R.-d.-l.-H.); jaime.tome@upm.es (J.T.-A.); rodrigolazarogorines@ucm.es (R.L.-G.); jggavila@ucm.es (J.G.G.); 2 Centre for Plant Biotechnology and Genomics (UPM-INIA), Universidad Politécnica de Madrid, Pozuelo de Alarcón, Madrid 28223, Spain

**Keywords:** immunotoxin, ribotoxin, α-sarcin, RNase T1, furin, intracellular trafficking, colorectal cancer

## Abstract

Immunotoxins are chimeric molecules that combine the specificity of an antibody to recognize and bind tumor antigens with the potency of the enzymatic activity of a toxin, thus, promoting the death of target cells. Among them, RNases-based immunotoxins have arisen as promising antitumor therapeutic agents. In this work, we describe the production and purification of two new immunoconjugates, based on RNase T1 and the fungal ribotoxin α-sarcin, with optimized properties for tumor treatment due to the inclusion of a furin cleavage site. Circular dichroism spectroscopy, ribonucleolytic activity studies, flow cytometry, fluorescence microscopy, and cell viability assays were carried out for structural and in vitro functional characterization. Our results confirm the enhanced antitumor efficiency showed by these furin-immunotoxin variants as a result of an improved release of their toxic domain to the cytosol, favoring the accessibility of both ribonucleases to their substrates. Overall, these results represent a step forward in the design of immunotoxins with optimized properties for potential therapeutic application in vivo.

## 1. Introduction

Due to their high cytotoxicity, specificity, and effectivity, immunotoxins have arisen as potent and promising antitumor agents [1,2,3]. These chimeric proteins are composed of a target domain that specifically targets a tumor marker, fused to a toxic domain, responsible for the cytotoxicity [4,5]. Their mechanism of action involves, in a first step, high affinity binding to the tumor antigen by the targeting domain, followed by the internalization of the complex by endocytosis, and finally the release of the toxic domain promoting the death of target cells [6,7,8]. Over the last years, multiple evidences have been gathered demonstrating that the efficiency of immunotoxins depends on different aspects [9,10] such as the following: antibody functional affinity and specificity for the tumor antigen expressed on the cell surface, the complex internalization efficacy, the rate of the toxin release in its intracellular route, and its intrinsic specificity and potency.

Currently, most of the immunotoxins are designed as recombinant fusion proteins. Different antibody fragments and linkers have been used. Thus, immunotoxins have been engineered to achieve greater specificity and tumor labelling and enhanced antitumor efficacy by reducing size and improving tumor penetration. Regarding this field, antibody engineering has pursued the development of different new formats with improved properties that are being incorporated within immunotoxin constructs [11,12,13,14]. Furthermore, beyond antibodies, other specific molecules, such as interleukins and growth factors, have been employed as targeting domains, leading to the first FDA-approved immunotoxins indicated for the treatment of acute myeloid leukemia [15] or hairy cell leukemia [16].

Toxins from different origins, have also been employed such as *Pseudomonas* exotoxin A, Diphteria toxin, actinoporins, gelonin, and the plant toxin ricin, among others [17,18,19,20,21,22,23,24,25,26]. Interestingly, ribonucleases (RNases) have acquired a significant importance due to their ideal features for being included as immunotoxin toxic domains [27,28,29,30,31,32]. In particular, ribotoxins stand out within the family of extracellular fungal RNases, as part of the toxic domain of immunotoxins, due to their small size, high thermostability and resistance to proteases, poor immunogenicity, and especially because they are highly effective to inactivate ribosomes [33,34,35,36,37,38]. As proven by the previous results obtained within α-sarcin-based immunotoxins, α-sarcin arises as the most promising ribotoxin to be included in these antitumoral therapeutic designs [36,38,39,40]. Its specific ribonucleolytic activity against just one single rRNA phosphodiester bond, located at the sarcin-ricin loop (SRL) of the larger rRNA, causes protein biosynthesis inhibition and apoptosis [41,42,43].

Few studies, however, have been focused on improving the effectiveness of immunotoxins by modulating their intracellular pathway [44,45,46]. As a general mechanism, once the target domain binds to the tumor antigen and gets internalized, the antigen-immunotoxin complex is found in the early endosomes, where it can be later recycled and presented back into the cell membrane or finally degraded into lysosomes. Toxin release and endosomal escape depends then on its intrinsic features. The two main routes that are usually followed by toxins are the following: (1) the route via the Golgi apparatus or (2) direct translocation to the cytosol [1]. Therefore, intracellular toxin trafficking can be considered to be a key checkpoint for desired cytotoxic effects and regarding cytotoxic efficiency, toxin delivery to the cytosol appears as a well-stablished rate-limiting step [1,47].

In this sense, we have previously produced and characterized two immunoconjugates, IMTXA33αS and scFvA33T1, based in the ribotoxin α-sarcin or the nontoxic RNase T1, respectively, fused to the variable domains (scFv) of the monoclonal antibody A33, which recognize and bind specifically the tumor-associated antigen GPA33, overexpressed in most of colorectal cancers [30,36,48,49]. We have characterized in detail both immunoRNases, not only for their structural and functional features, but also as a model to evaluate the effect of the different toxic domains and the relationship between intracellular trafficking and immunotoxins cytotoxicity [39,40].

**Figure 1:**
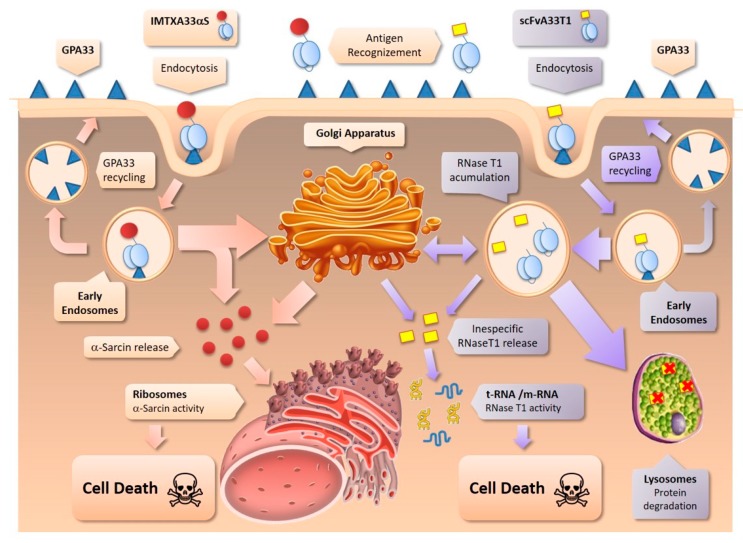
Scheme of the intracellular route followed by IMTXA33αS and scFvA33T1. As previously described [37], IMTXA33αS is internalized via early endosomes and follows the Golgi apparatus retrograde pathway, before α-sarcin release to the cytosol to exert its ribonucleolytic activity. Once internalized, scFvA33T1 appears also in the Golgi apparatus but mainly it is driven to lysosomes. These different pathways explain the more cytotoxic efficiency of IMTXA33αS.

Briefly, the antitumoral activity differences observed between both constructs have been explained by two aspects. The exquisite specificity of the ribonucleolytic activity of α-sarcin against ribosomes [36,40] in comparison with that exhibited by RNase T1 [30,50] and the intracellular pathway followed by each toxic domain, being the latter extremely decisive [39]. On the one hand, regarding the enzymatic properties of RNase T1, it is a much less specific acid cyclizing ribonuclease, with preference for the hydrolysis of GpN bonds. Although it has the same catalytic mechanism as ribotoxins, the latter present structural differences and small modifications in their catalytic residues that make them highly specific in terms of their ribonucleolytic activity. On the other hand, α-sarcin release to the cytosol could be carried out directly from endosomes or from the retrograde pathway involving Golgi apparatus, due to its ability to interact with the acidic components of the endosomes and Golgi membranes. Conversely, RNase T1, a nontoxic RNase, with an acidic isoelectric point value (pI), is not able to interact with the acidic components of endosome or Golgi membranes. Therefore, its release into the cytosol is impaired, favoring its degradation in the lysosomes or its accumulation into the late Golgi apparatus (Figure 1) [39].

In this context, some designs of immunotoxins with linkers including a specific furin cleavage site have been shown to be more cytotoxic [51,52,53,54]. This observation would confirm that intracellular processing and release of the toxic moiety is one of the key optimization spots for immunotoxin design [55,56,57]. Furin is a transmembrane enzyme present in the plasma membrane, the endosomes, and most notably in the trans-Golgi network [58]. It is a serine protease that belongs to the subtilisin family. Furin was known as the first mammalian proprotein-processing enzyme, exhibiting cleavage specificity for paired basic amino acid residues. Proteins were cleaved just downstream of the target sequence, canonically, Arg-X-(Arg/Lys)-Arg’, with a minimal recognition cleavage site, described as Arg-X-X-Arg [59,60].

**Figure 2:**
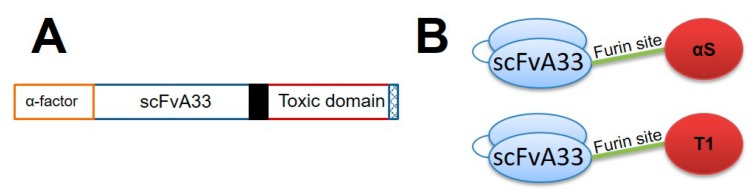
Schemes showing both genetic and domain structures of scFvA33-furin based immunotoxins. (**A**) Diagrammatic representation of gene construct. Both constructs include the α-factor signal peptide for secreted expression in *P. pastoris*, the anti-GPA33 scFvA33 gene (VH-linker-VL), a furin cleavage site linker (black box), the toxic domain, and the six His-tag (hatched box). The toxic domain was formed by ribotoxin α-sarcin or RNase T1. (**B**) Schematic model of the domain structure of scFvA33-furin based α-sarcin (upper) or RNase T1 (lower) immunotoxins.

Within this idea, in this work we describe the production, purification, and in vitro functional characterization of two colorectal antitumor immunoRNases, including a furin cleavage site, based in the fungal ribotoxin α-sarcin and RNaseT1, IMTXA33furαS and scFvA33furT1, respectively (Figure 2). It is noted that our immunotoxins are usually produced in the heterologous system *P. pastoris*, a generally regarded as safe (GRAS) organism [61]. Thus, the immunotoxin secretion to the extracellular medium is driven by the α-factor signal peptide, which is finally released from the mature immunotoxin polypeptide chain by the action of Kex2 proteases. As furin belongs to this family and exhibits some of their recognition site characteristics, a minimum recognition sequence of furin has been used to avoid recognition by the Kex2 protease present in *P. pastoris* [62].

## 2. Results

### 2.1. IMTXA33furαS and scFVA33furT1 Variants Were Purified as Fully Functional Immunoconjugates

**Figure 3:**
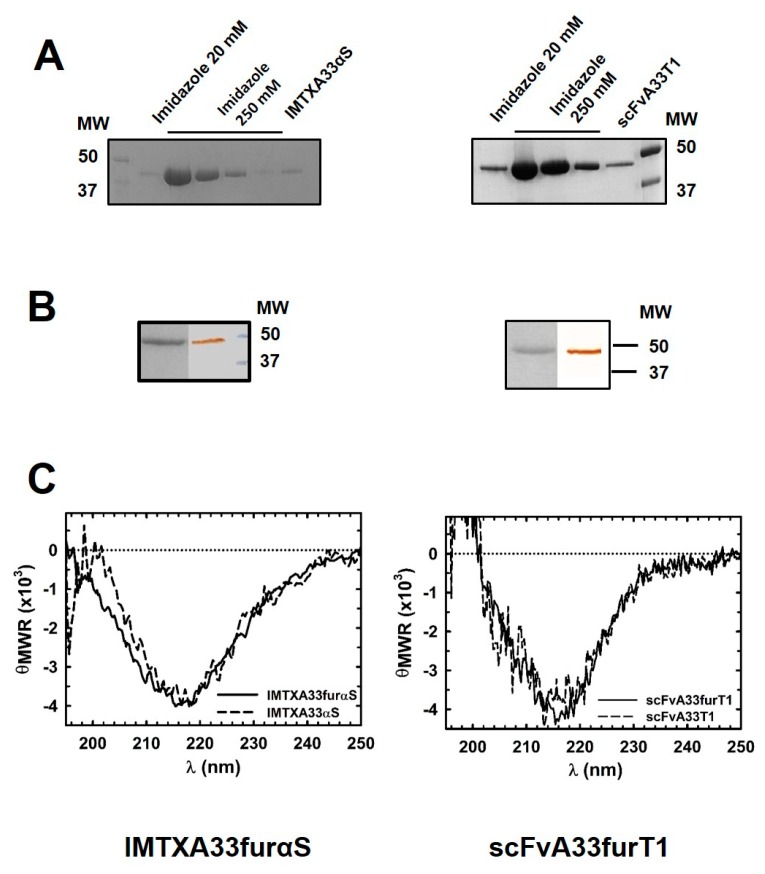
Production, purification, and structural characterization of furin-variant immunotoxins: IMTXA33furαS (left) or scFvA33furT1 (right). In both cases: (**A**) SDS-PAGE analysis of aliquots taken from IMAC performed to purify IMTXA33furαS or scFvA33furT1, visualized by Coomassie brilliant blue staining. (**B**) Purified IMTXA33furαS or scFvA33furT1 final fraction detection by Coomassie brilliant blue (left lane) or western blot (right lane). MW corresponds to prestained protein standards (Bio-Rad). (**C**) Far-UV circular dichroism spectra (θ_MRW_, mean residue weight ellipticities were expressed as degree × cm^2^ × dmol^−1^) of: IMTXA33furαS (solid line) and IMTXA33αS (short dash line) (left); scFvA33furT1 (solid line) and scFvA33T1 (short dash line) (right).

Both IMTXA33furαS and scFVA33furT1 variants were successfully produced in the extracellular media of *P. pastoris* cultures after 48 h of methanol induction. Then both immunoconjugates were purified following dialysis and immobilized metal affinity chromatography (IMAC) (Figure 3A), as described in the Methods section. SDS-PAGE and western blot immunodetection, under reducing conditions, were carried out to analyze the identity and homogeneity of the purified proteins (Figure 3B). Bands of 42 and 45 kDa for scFvA33furT1 and IMTXA33furαS, respectively, corresponding to the expected theoretical molecular weight, were visualized by Coomassie blue staining and were also recognized by the anti-α-sarcin serum or anti-His-tag antibody. The final purification yield for both proteins was 1.5 and 3 mg per liter of induction medium for IMTXA33furαS and scFVA33furT1, respectively. The far-UV CD spectra for both IMTXA33furαS and scFvA33furT1 were coherent with those expected, according to the structural features of the domains that conform both immunoconjugates, and therefore compatible with water-soluble globular functional proteins. In this sense, a high content in beta sheet was observed that can be attributed to the folding described for the scFvA33, as well as to the contribution of RNase T1 and α-sarcin native conformation, which also present a high content of β-sheet and a small contribution of alpha helix [30,36] (Figure 3C).

**Figure 4:**
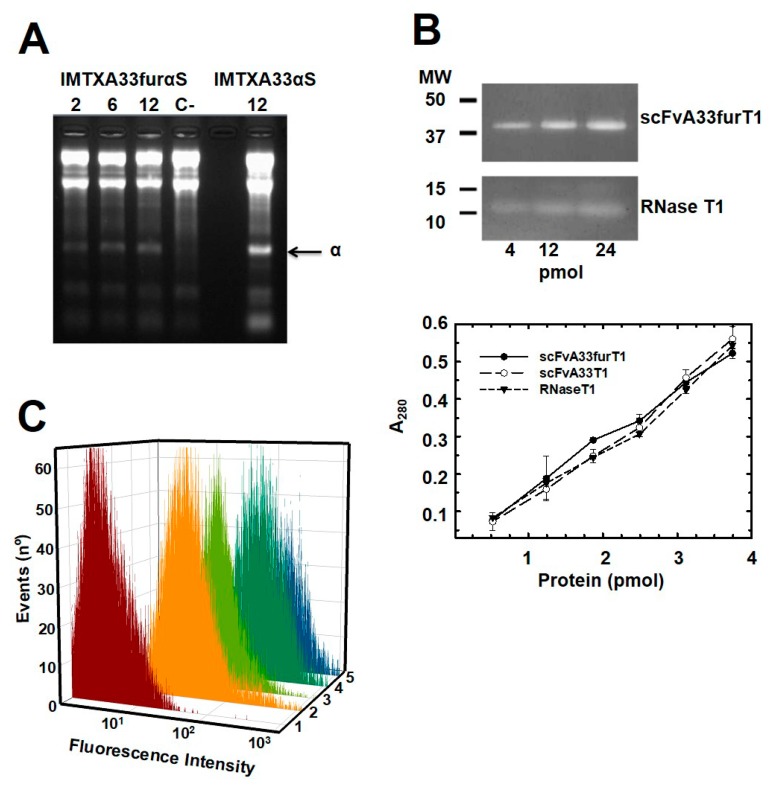
Functional characterization: (**A**) RNase activity assay of purified IMTXA33furαS using rabbit ribosomes as substrate. The characteristic α-fragment, as product of the specific RNase activity of α-sarcin is shown (indicated by an arrow). For IMTXA33furαS, 2, 6, and 12 pmol were assayed and 12 pmol of IMTXA33αS were used as a control. (**B**) RNase activity assays of purified scFvA33furT1. Poly(G) zymogram assay (upper panel) after SDS-PAGE of scFvA33furT1 or RNase T1. Four, 8 and 12 pmol of protein were assayed. Colorless bands were a consequence of the RNase T1 unspecific ribonucleolytic activity. MW (kDa), corresponds to electrophoretic molecular mass markers. Yeast RNA degradation assay (lower panel): The graph represents the A_260_ values versus the different amounts of scFvA33furT1 (black circles), scFvA33T1 (open circles), and RNase T1 (triangles) assayed. (**C**) Flow cytometry analysis of scFvA33furT1 (plot 2), scFvA33T1 (plot 3), IMTXA33furαS (plot 4), and (IMTXA33αS (plot 5) binding to SW1222 (GPA33-positive cells). The control curve (plot 1) corresponds to cells just treated with the anti-Histag-Alexa 488 antibody (Santa Cruz Biotechnologies, Santa Cruz, CA, USA).

To assess the correct functionality of these furin-variants immunoconjugates we first analyzed the ribonucleolytic activity of the two RNases included in their toxic domains. As expected, IMTXA33furαS was able to release the characteristic α-fragment, due to the specificity of its ribonucleolytic activity against the rRNA sarcin-ricin loop (SRL) present in the ribosomes (Figure 4A). Regarding scFvA33furT1, it exhibited nondistinguishable ribonucleolytic activity as compared with RNase T1 wild type or scFvA33T1 immunoconjugate when assayed in zymogram analysis against its typical substrate, poly(G), and in solution when *Torula* yeast RNA hydrolysis was measured (Figure 4B). The highly acidic feature of RNase T1 and its smaller size as compared with the immunoRNase, might explain the different intensities of the bands corresponding to scFvA33T1 and RNase T1. Thus, the incubations required to perform the assay would allow it to leak out of the gel. Both assays revealed proper functionality of the RNase moiety in the scFvA33furT1 variant. The ability of the targeting domain from both immunoconjugates to bind GPA33-positive cells SW1222 was also evaluated. As determined by flow cytometry results, both furin-variant constructs recognized GPA33-positive SW1222 cells (Figure 4C) as efficiently as IMTXA33αS and scFvA33T1, used as controls.

**Figure 5:**
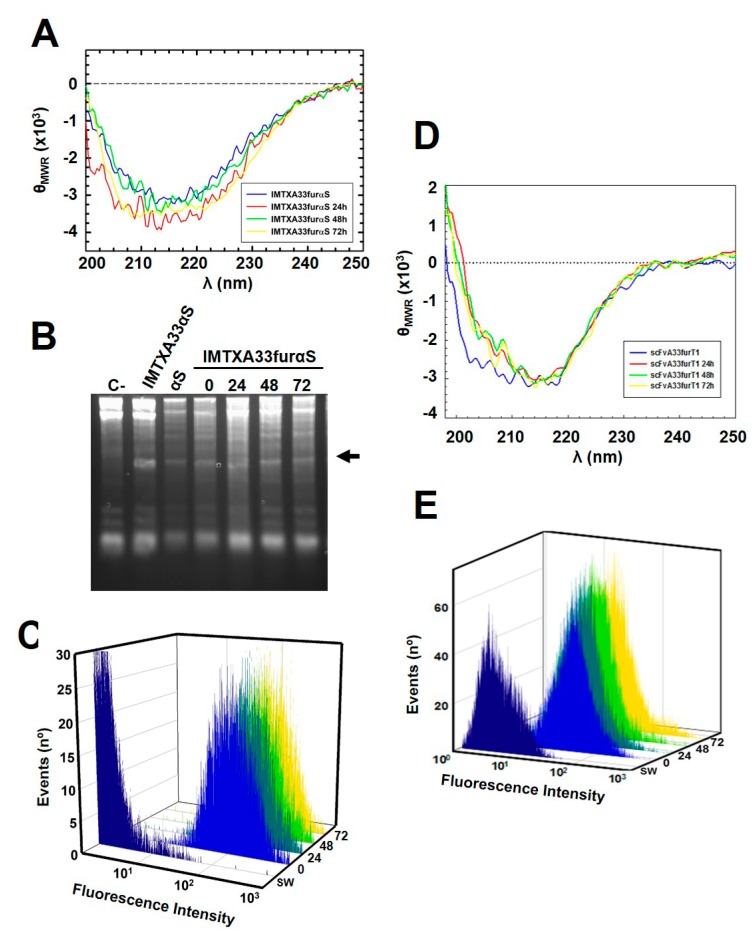
Stability assays of IMTXA33furαS (**A**–**C**) and scFvA33furT1 (**D**–**E**): In all cases, samples were previously incubated at 37 °C for 0, 24, 48, and 72 h. (**A**,**D**) Far-UV circular dichroism spectra (θ_MRW_, mean residue weight ellipticities were expressed as degree × cm^2^ × dmol^−1^). Spectra were made with protein at 0.15 mg/mL in RPMI 1640 medium. (**B**) Specific ribonucleolytic activity against rabbit ribosomes. Samples were previously incubated at 37 °C in the absence of the substrate. The appearance of the α-fragment was indicated by an arrow. IMTXA33αS and α-sarcin wild type were used as controls. In all cases, 6 pmol of protein were assayed. (**C**,**E**) Binding assay o GPA33-positive SW1222 cells by flow cytometry analysis. Control curves (as in Figure 4).

Moreover, both constructs showed high structural stability and also maintained their functional integrity, when conditions mimicking a physiological context were assayed. In this sense, when purified proteins were incubated in media for at least 72 h at 37 °C, far-UV CD spectra showed that the full molecular folding of both immunotoxins were kept (Figure 5A,D). In addition, in the same conditions and for at least 72 h, the specific ribonucleolytic activity of α-sarcin (Figure 5B) and the specific antigen-binding ability of the target domain, in accordance with the flow cytometry analysis, were also preserved (Figure 5C,E).

### 2.2. IMTXA33furαS and scFVA33furT1 Follow Different Intracellular Pathway

Once it was established that the new immunoconjugate variants kept intact their antigen-specific binding capacity, as well as their ribonucleolytic activities, the next step was to analyze the effect of the furin cleavage site on the intracellular pathway followed after their internalization. In order to study this aspect, first the new constructs were labeled with Alexa 555. Both modified proteins remained fully structured, in comparison with the unlabeled versions (Figure S1), according to their far-UV CD spectra.

**Figure 6:**
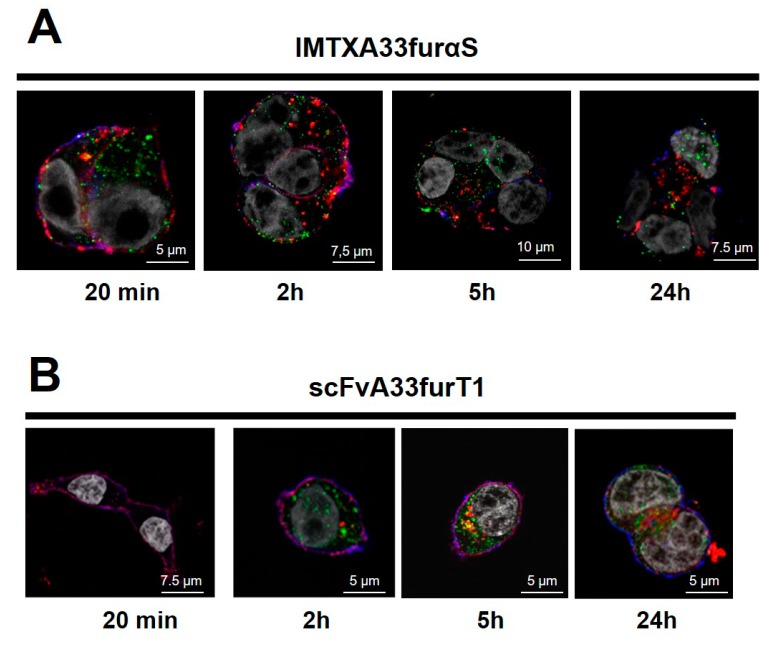
Endosomes colocalization analysis: Immunofluorescence confocal microscopy images obtained from cells incubated with IMTXA33furαS-555 and scFvA33furT1-555. Immunofluorescence confocal microscopy images of SW1222 cells after incubation for 20 min, 2 h, 5 h, or 24 h (from left to right) with IMTXA33furαS-555 (**A**) or scFvA33furT1-Alexa 555 (**B**). In all cases, images correspond to merging of nuclei labeled with DAPI (grey), plasmatic membrane were visualized with anti-CD44 plus GAM-Alexa 647 (blue), early endosomes were visualized with anti-EEA1 plus GAR-Alexa 488 (green), IMTXA33furαS-555 or scFvA33furT1-555 (red). The appearance of yellow dots indicates colocalization with endosomes, while violet dots correspond to colocalization with the plasmatic membrane.

Internalization and intracellular localization of both labeled constructs were then studied. As shown for IMTXA33furαS (Figure 6A) and scFvA33furT1 (Figure 6B), very low colocalization was observed for both immunoconjugates with early endosomes. Although, no substantial differences were found between both constructs, interestingly, IMTXA33furαS colocalization was detected at shorter incubation times, 20 min or 2 h, than that observed for scFvA33furT1, at 5 or 24 h.

**Figure 7:**
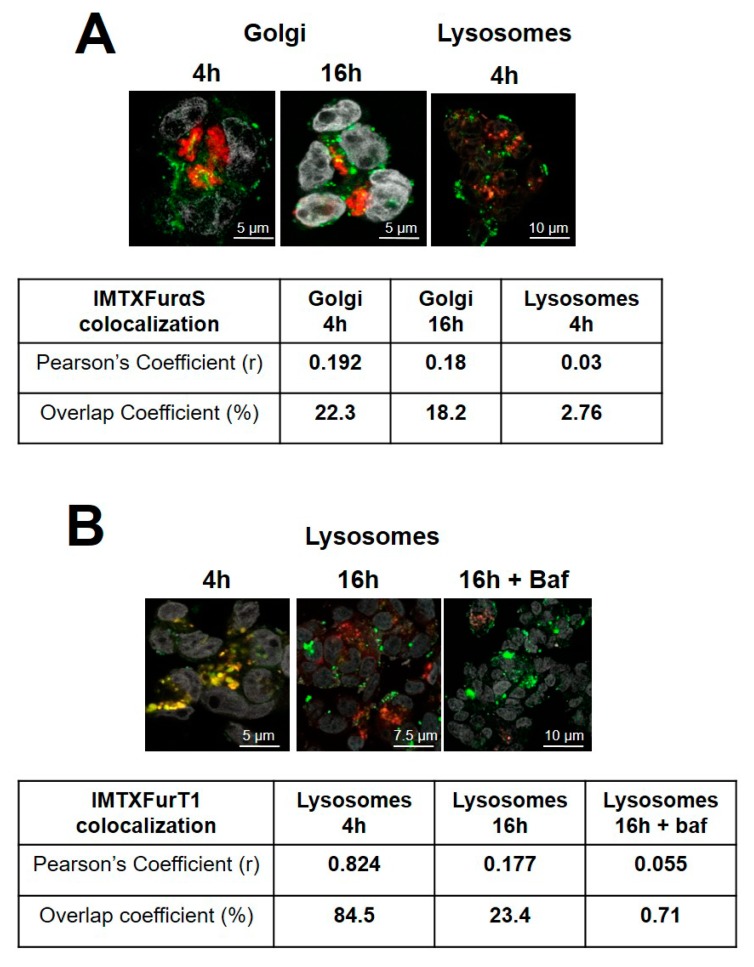
Golgi apparatus and lysosomes colocalization analysis: Immunofluorescence confocal microscopy images obtained from cells incubated with IMTXA33furαS-Alexa-555 (**A**) or scFvA33furT1-Alexa-555 (**B**). (**A**) SW1222 cells were incubated for 4 or 16 h with both immunoconjugates as indicated in the figure. In all cases, images correspond to merging of nuclei labeled with DAPI (grey), Golgi with agglutinin or lysosomes with Lysotracker (red) and IMTXA33furαS-555 or scFvA33furT1-555 (green). (**B**) In the case of scFvA33T1-555, incubation was also made adding bafilomycin at 5 ng·mL^−1^. Quantitative colocalization analysis was made as indicated in Methods. The appearance of yellow dots indicates colocalization with Golgi (**A**) or lysosomes (**B**).

As previously described for IMTXA33αS and scFvA33T1, two main intracellular pathways are followed by the toxic domain once the endosomes have been reached, via lysosomes or via Golgi-apparatus. On the one hand, for IMTXA33furαS, partial colocalization degree (22%, overlap coefficient) with Golgi apparatus at 4 h was observed, remaining at similar levels after 16 h of incubation (18%) (Figure 7A). However, colocalization with lysosomes was almost negligible. On the other hand, taken into account that for RNase T1-based immunoconjugates a significant colocalization with lysosomes was previously described [39], lysosome colocalization for scFvA33furT1 was measured. In this case, a significant colocalization was observed at 4 h, with degrees of 84%, but was dramatically decreased after 16 h (23%), and nearly disappeared when cells were treated with the antibiotic bafimolycin (<1%) (Figure 7B).

The results obtained with the furin variants, compared to those described previously for the original constructions, show significant differences in the degree of colocalization with the cellular organelles involved in their intracellular pathways. On the one hand, in the case of IMTXA33furαS, although it follows the endosomas-Golgi route, just like the original construct [39], its detection in endosomes and Golgi apparatus is significantly lower, suggesting a greater or better release to the cytosol. On the other hand, for scFvA33furT1, there is a very significant increase in colocalization with the Golgi and a decrease in the case of lysosomes. As later discussed, these results would suggest that the presence of the furin cleavage site facilitates the release to the cytosol of the toxic domain of both immunoconjugates.

### 2.3. The Presence of the Furin Cleavage Site Significantly Increases the Cytotoxicity of Both Immunoconjugates

**Figure 8:**
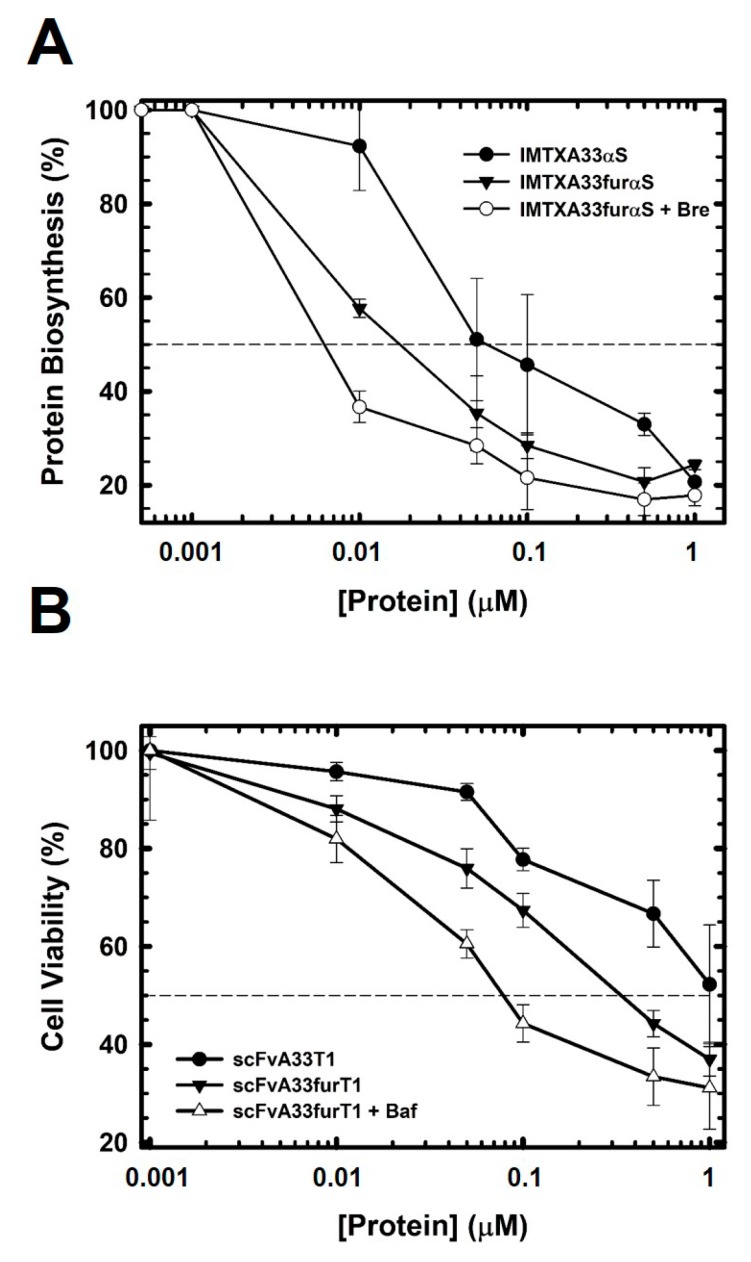
Cytotoxic characterization of IMTXA33furαS (**A**) and scFvA33furT1 (**B**). (**A**) Protein biosynthesis inhibition assay. SW1222 cells were incubated for 72 h with IMTXA33αS (●), IMTXA33furαS (▼), and IMTXA33aS + brefeldin A (○). (**B**) MTT viability assay of SW1222 cells incubated for 72 h with scFvA33T1 (●), scFvA33furT1 (▼), and scFvA33furT1+ bafilomycin (Δ). The 50% level of protein biosynthesis inhibition or cell viability were indicated (dashed line).

In vitro cytotoxicity assays were performed, to assess the effect of furin cleavage at the specific site included in these new designs. IMTXA33furαS showed an enhanced cytotoxicity with an IC_50_ two-times lower than that described for IMTXA33αS (Figure 8A). Even more interesting, cytotoxicity was increased by an additional three times when brefeldin A, that causes disassembly of Golgi-apparatus, was added (Figure 8A). However, the addition of bafilomycin that inhibits lysosomal acidification, resulted in no change in the cytotoxicity of this construction (data not shown). On the other hand, scFvA33furT1 toxicity was increased up to three times as compared to that described for scFvA33T1, being even greater when bafilomycin was added (Figure 8B). In this case no significant effect was observed with brefeldin A (data not shown).

## 3. Discussion

We have purified and characterized two new optimized immunoconjugates, IMTXA33furαS and scFvA33furT1, which include a specific cleavage site for furin in the linker between the targeting and toxic domains of both constructs. They were purified to homogeneity from *P. pastoris* cultures, showing the expected size and folding, according to their biophysical characterization. This structural characterization yielded results which were consistent with the features previously described for their original counterparts, IMTXA33αS and scFvA33T1 [30,36], results that also were fully consistent, regarding to the nature and content of the secondary structure elements described for α-sarcin, RNase T1, and A33 scFv, their native domains [63,64,65,66,67]. Both constructs were also able to specifically bind to GPA33 expressed on the surface of the membrane and be internalized into SW1222 colorectal cancer cells, indicating that the targeting domain was fully functional.

Furthermore, they kept the original α-sarcin and RNase T1 ribonucleolytic activities, needed for ribosome inactivation and RNA hydrolysis, respectively. Moreover, when physiological-like conditions were analyzed, both IMTXA33αS and scFvA33T1, were stable and functional. In this sense, we could conclude that both immunoconjugates were correctly folded in terms of their structural and functional features, at least to the same extent as their original designs, IMTXA33αS and scFvA33T1.

As mentioned before, there is a close relationship between the intracellular route followed by the proteins included in the toxic domains of immunoconjugates and the antitumor efficiency of the immunotoxins [1]. Accordingly, this relationship had been previously reported for IMTXA33αS and scFvA33T1 [39]. Thus, once internalized, IMTXA33αS mainly followed the endosome-Golgi-apparatus network, whereas scFvA33T1 appeared indistinctly distributed between the lysosomes and the Golgi-apparatus. It is precisely this difference in the internalization pathway followed that led to a greater cytotoxic efficiency of IMTXA33αS [39].

Different designs of immunotoxins have been described including linkers with a furin cleavage site showing an increase in cytotoxic efficacy [51,52,53,54], confirming that intracellular processing and release of the toxic moiety is one of the key optimization spots in immunotoxin design [55,56,57]. Within this context, the main purpose driving this work was to evaluate the effect of including a furin linker in the previously characterized α-sarcin- and RNaseT1-based immunoconjugates [30,36,39]. The starting hypothesis was that this protease-specific site would ease the toxic moieties release and, consequently, would improve the cytotoxic efficiency of IMTXA33furαS and scFvA33furT1. Quite surprisingly, and although the results obtained for both constructs confirmed the preferred intracellular route used by each of the constructs [39], they, nevertheless, showed significant differences from what had been described for their parental designs.

The results herein presented further confirm that the cytotoxic mechanism of α-sarcin-based immunotoxins, IMTXA33furαS and IMTXA33αS, involves the endosome-Golgi intracellular pathway. The differences observed between both immunotoxins concerning the colocalization rate with the Golgi apparatus are in good agreement with their cytotoxic efficiency (Table 1). IMTXA33furαS cytotoxicity was two-fold higher than that observed for IMTXA33αS. Interestingly, the furin variant and its parent counterpart exhibited completely different behavior when traffic through the Golgi was blocked by the addition of brefeldin A. Whereas in the case of the original immunotoxin its cytotoxicity was dramatically reduced as an effect of brefeldin A, for the furin variant the cytotoxicity was significantly increased. These differences can only be explained by the presence of the furin cleavage site in the design of the immunotoxin. Furin protease activity would produce an increase in the amount of free α-sarcin that can interact with endosomal membranes and thus be released more efficiently to the cytosol, even without reaching the Golgi apparatus. It has been proven a long time ago that wild-type α-sarcin has the ability to translocate across a lipid bilayer rich in negatively charged phospholipids [68]. However, in the case of the original immunotoxin, most of the chimeric protein would reach the Golgi, and therefore the action of brefeldin A would negatively affect the release of the toxin. It must be emphasized that the inner membranes from both endosomes and Golgi apparatus have a high content of negative charges [69,70,71,72], contributing to α-sarcin translocation to the cytosol directly from endosomes or from Golgi apparatus [42,43]. Moreover, furin exert its protease activity not only in the Golgi but also in the endosomes [58]. Thus, α-sarcin release to the cytosol is facilitated by the presence of the furin cleavage site.

For scFvA33furT1, significant enhanced cytotoxicity was also observed when compared to the original design, scFvA33T1. As observed in the internalization assays, the degree of colocalization with lysosomes was significantly lower when compared to that previously described for scFVA33T1 [39] (Table 1), suggesting that most of scFvA33fur1 could follow the Golgi pathway or that the inclusion of the furin cleavage site would again favor its accumulation in endosomes. Within this, addition of bafilomycin, which impaired the recycling in the lysosomes, produced and increased in the cytotoxic effectiveness. However, the best IC_50_ value obtained for scFvA33furT1 (70–90 nM) after incubation with bafilomycin did not improve the cytotoxic effectiveness obtained by its original equivalent in the presence of the same antibiotic. Regarding this, it must be noted that the acidic pI value of RNase T1 [50,73] hamper its interaction with the endosome or Golgi membranes. Even if the lysosome pathway was partially blocked, the release into the cytosol was impaired. In fact, there is not any published account of RNase T1 being able to translocate a biological membrane on its own.

In summary, our results confirm the different routes followed by α-sarcin and RNaseT1 when included as part of the toxic domain of an immunotoxin and how we can take advantage of their features including a furin cleavage site in its design. Both furin-variant immunoconjugates exhibit enhanced antitumor effectiveness than that described for their original parents. Therefore, in vivo assays with IMTXA33furαS, exhibiting increased antitumor activity than that based in RNase T1, must be addressed.

## 4. Conclusions

This work not only represents a step forward in optimizing the cytotoxic efficacy of immunotoxins based on α-sarcin and RNase T1, but also highlights the development of an immunotoxin design platform based on these ribonucleases, including new designs with different specificities, with monomeric or trimeric formats [74,75], or including the use of non-immunogenic variants of α-sarcin [38]. The combination of all these optimization approaches will represent a very important boost for its application in the clinic.

## 5. Materials and Methods

### 5.1. Plasmid Design

Plasmids encoding IMTXA33αS and scFvA33T1 were previously obtained [30,36]. PCR was used to amplify the cDNA sequences of interest, using the necessary oligos to incorporate the sequence coding for the furin cleavage site between the target and the toxic domains, and also the restriction sites needed for cloning. Once purified, the resultant IMTXA33furαS and scFvA33furT1 cDNAs were cloned in pPICZαA (Invitrogen), for their expression in the methylotrophic yeast *P. pastoris KM71*. To facilitate protein detection and purification, as in their original counterparts, a 6 His-tag was included at the C-terminus of both immunotoxins (Figure 2). The expression vectors were amplified in *E. coli DH5αF’* and subsequently sequenced, using the DNA sequencing service of Universidad Complutense’s Genomics Unit.

### 5.2. Protein Production and Purification

Electrocompetent *P. pastoris KM71* cells were used to electroporate 5–10 µg of the corresponding linearized plasmid previously cleaved with *Pme* I. A Bio-Rad Gene pulser apparatus (Bio-Rad, Berkeley, CA, USA) was used for this purpose. Multiple independent clones were selected with different amounts of zeocin (100, 400, or 750 µg/mL) and assayed to find the most productive colonies. To carry out these screenings, cells were grown in BMGY in 24-well plates at 30 °C for 24 h, were harvested afterwards, and finally suspended in BMMY. For induction of protein production, the cuture was shaked at 25 °C and 200 rpm, as previously described [30,36,39].

Once the induction was completed, the secretion to the extracellular media of the proteins of interest was analyzed by 0.1% (*w*/*v*) sodium dodecyl sulfate (SDS)-15% (*w*/*v*) polyacrylamide gel electrophoresis (PAGE) and western blot. In this sense, for the specific detection of the toxic domain by western blot, a rabbit anti-α-sarcin serum was used. In addition, an anti-histidine tag antibody was used to check integrity of the purified proteins. Large-scale production of both immunotoxins was carried out by addition of 25 mL of preinoculum to a 2 L baffled flasks containing 350 mL of BMGY. Then the culture was incubated with shaking for 16 h at 30 °C and 220 rpm. Cells were recollected by soft centrifugation at room temperature and resuspended again in 200 mL of BMMY. For induction of protein production, cells were incubated at 25 °C, 250 rpm shaking, for 48 h, supplementing the culture with methanol every 24 h. Once finalized, dialysis of the extracellular medium containing the proteins of interest was carried out, using a 50 mM sodium phosphate buffer with 0.1 M NaCl, pH 7.5.

IMTXA33furαS and scFvA33furT1 were purified from the dialyzed extracellular medium by IMAC. A Ni^2+^-NTA agarose column was used for this purpose (HisTrap™ FF Columns, GE Healthcare, Fairfield, CT, USA). The extracellular media was applied to the column at 1 mL/min. The flow rate was controlled by a peristaltic pump. Two consecutive washes were done with the dialysis buffer and with the same but adding 20 mM imidazole, before elution of the proteins of interest, by using the same buffer but containing 250 mM imidazole. Finally, the different aliquots containing the purified proteins were collected and dialyzed again against the dialysis buffer.

### 5.3. Biophysical Characterization

Absorbance measurements were performed on an Uvikon 930 spectrophotometer (Kontron). As described before [76], far-UV circular dichroism (CD) spectra were carried out using a Jasco 715 spectropolarimeter. Proteins dissolved in PBS were prepared at 0.15 mg/mL. Cells of 0.1 cm optical path were used. Four spectra were averaged to obtain the final data. For stability assays, spectra were obtained after previous incubation of both constructs at 37 °C for 0, 24, 36, 48, or 72h in RPMI 1640.

### 5.4. Ribonucleolytic Activity Assays

The specific ribonucleolytic activity of α-sarcin, included in the toxic domain of IMTXA33furαS, was followed as described before [73,77]. For detection of the release of the characteristic 400 nt rRNA, namely α-fragment, we used as substrate ribosomes from a rabbit cell-free reticulocyte lysate. Briefly, the lysate was diluted three-fold in 40 mM Tris-HCl buffer, pH 7.5, containing 40 mM KCl and 10 mM EDTA. Then, 50 μL aliquots were taken of this dilution (5–6 pmol of ribosomes approximately) and incubated for 15 min at room temperature with different amounts of the proteins to be assayed. Then, the reaction was finished by adding 250 μL of 50 mM Tris-HCl, pH 7.4, 0.5% (*w*/*v*) SDS, followed by briefly vortex. Subsequently, RNA phenol/chloroform extraction was carried out. The RNA pellet obtained by the addition of isopropanol to the aqueous phase, was then washed with 70% (*v*/*v*) ethanol, dried exhaustively, and finally resuspended in 10 μL of DEPC H_2_O. The presence of α-fragment in the samples was detected by ethidium bromide staining after electrophoresis, on denaturing 2% agarose gels. For quantification of the bands we used the Gel Doc XR Imaging System and Quantity One 1-D analysis software (Bio-Rad, Berkeley, CA, USA). Original IMTXA33αS [36] was used as the control.

The ribonucleolytic activity of RNase T1, included in scFvA33furT1, was analyzed by two parallel assays. First, RNA degradation in solution was tested by measurement of *Torula* yeast RNA (Sigma, type VI; Sigma-aldrich, St. Louis, MI, USA) hydrolysis [73]. In this assay the absorbance values obtained are proportional to the amount of soluble small oligonucleotides resulting from the RNasa activity on RNA larger size fragments contained in the substrate sample. The results obtained for the RNA samples analyzed (2 mg/mL) without protein were used to consider nonenzymatic RNA degradation, while original scFvA33T1 [30] and free RNase T1 were used as controls. In the second assay, the activity of purified scFvA33furT1 and RNase T1 was evaluated following the zymogram method [78]. Briefly, samples were applied into SDS-PAGE in 15% polyacrylamide gels, containing 0.1% (*w*/*v*) SDS and embedded with 0.3 mg/mL poly(G) homopolyribonucleotide [79]. Proteins exhibiting RNase activity were visualized as colorless bands, whereas the rest of the gel appeared colored.

### 5.5. Cell Lines Culture

Colon carcinoma SW1222 cells were used as model for the GPA33-positive cellular line [30,36,39]. The cultures were grown as described [30], in RPMI 1640 medium (Sigma-aldrich, St. Louis, MI, USA), supplemented with glutamine (300 mg/mL), containing 50 U/mL of penicillin and 50 mg/mL of streptomycin. Finally, the media was supplemented with 10% fetal bovine serum (FBS). Incubation of the cells was carried out at 37 °C in a humidified atmosphere (CO_2_:air, 1:19, *v*:*v*). Trypsinization was routinely done for harvesting and propagation of the cultures. A hemocytometer was used to count the cells used in all assays described.

### 5.6. Flow Cytometry Studies

Trypsinized cells were distributed into different aliquots, containing 3 x 10^5^ cells/mL, and washed several times with PBS 0.1% (*w*/*v*) BSA containing 0.02% (*w*/*v*) sodium azide. The samples were incubated with gentle shaking for 1 h at room temperature, with 1 μM of IMTXA33furαS or scFvA33furT1, using their original counterparts as positive controls. A second incubation, adding anti-His-Alexa488 (Sigma-aldrich, St. Louis, MI, USA) diluted 1/100, was carried out in the dark. When necessary, between the different steps, cells were collected by centrifugation (1200× *g*, 4 °C, 10 min) and then washed with PBS for several times. Flow cytometry acquisition was done on a FACScan (Becton Dickinson, Becton Dickinson, NJ, USA) and data were obtained using the WinMDI software. For stability assays, IMTXA33furαS and scFvA33furT1 were previously incubated at 37 °C for different times (0, 24 or 72 h).

### 5.7. Fluorescence Microscopy

To carry out these assays, both immunotoxins were first labeled. As previously described [37], using the Alexa Fluor 555 Protein Labeling Kit (Invitrogen, Carlsbad, CA, USA). Both labeled conjugates (IMTXA33furαS-555 and scFvA33furT1-555) were purified and characterized to ensure the preservation of their structural and functional features.

SW1222 cells were first trypsinized, seeded at 8 × 10^5^ cells/well using cover-glasses, and incubated overnight at 37 °C. For treatment, IMTXA33furαS-555 or scFvA33furT1-555, at 25 µg/mL, were added to cells for the following different periods of time: from 20 min to 24 h for endosomes colocalization assays, and 4 or 16 h when lysosomes/Golgi colocalization assays were performed. Confirmation of colocalization with lysosomes was also studied by the addition of bafilomycin at 5 ng/mL, an antibiotic that inhibits lysosomal acidification [80], in combination with the immunoRNase. To observe the plasmatic membrane, incubation with anti-CD44 mAb [81] were performed. After removing the medium, the cells were fixed for 15 min with PBS containing 3% (*v*/*v*) p-formaldehyde and incubated for 15 min in PBS containing 50 mM ammonium chloride. Cells were permeabilized by the addition of digitonine at 0.01% (*w*/*v*) in PBS and incubation for 30 min, followed by another incubation in PBS containing 1.0% (*w*/*v*) BSA for 1 h. Different probes were used for organelle labeling as follows: an Ab targeting protein EAA1, present in the early endosomes [82]; lysosomes were labeled with Lysotracker (Life Technologies, Carlsbad, CA, USA), a fluorescence acidotropic specific probe [83]; and Golgi apparatus with Wheat Germ Agglutinin (Life Technologies) that binds to the highly abundant sialic acid present in the Golgi membranes [84]. Donkey anti-mouse Alexa 647 (DAM-Alexa 647) or goat anti-rabbit Alexa 488 (GAR-Alexa 488) were also added as secondary antibodies. For nuclei labeling, 10 µL of Prolong Gold-DAPI (Life Technologies) were added. Incubations were carried out at room temperature and the final samples were kept at 4 °C. To obtain the corresponding images, a Leica TCS SP2 confocal microscope was used, followed by analysis with the LCS lite software. Images presented correspond to the optical planes concerning the internal content of the cells selected for analysis. For each sample, ten different images were taken along the Z-axis, covering all the cells, from the basal zone to the apical one, Images shown correspond to the internal content of the cells, in particular to slices 4 to 6. ImageJ software was used to performed colocalization quantification, using two coefficients, the Pearson’s correlation coefficient and, as described by Mander, the overlap coefficient. Both coefficients referred to the correlation between the intensity distribution of the channels and the true degree of colocalization, respectively [85].

### 5.8. MTT Viability Assay

Cell viability was evaluated by using the MTT-Cell Proliferation Kit I (Roche, Basel, Switzerland) as previously described [30,39]. Briefly, 5 × 10^3^ trypsinized cells/well were seeded and then incubated for 24 h at 37 °C. The medium was then removed and scFvA33furT1 or scFvA33T1 were added at different concentrations in 200 µL final volume. Samples where incubated for 96 h, followed by another incubation with MTT at 0.5 mg/mL during 4 h at 37 °C. Finally, the solubilization buffer was added and the viability was determined in terms of A_595_ nm, whereas the higher A_595_ values were in correspondence with increasing amounts of viable cells. Cells incubated only with medium, in the absence of the protein, were taken as 100% viability. If necessary, samples including bafilomycin at 5 ng/mL, together with the protein were also done. Control only with bafilomycin, was included to evaluate potential drug related toxicity. The results shown correspond to the average of four independent assays.

### 5.9. Protein Biosynthesis Inhibition

To evaluate the cytotoxicity of ribotoxins the protein biosynthesis inhibition assay is routinely used [41,42,43]. Briefly, cells were seeded into 96-well plates (1 × 10^4^ cells/well) in culture medium and kept under standard culture conditions for 24 h. Different concentrations of the different immunoconjugates, in 200 µL of free-FBS fresh medium were added to the cells. After 72 h of incubation at 37 °C, the medium was removed, followed by replacement with a fresh one containing 1 mCi per well of L-[4-C-3H]-Leucine (166 Ci/mmol; GE Healthcare, Fairfield, CT, USA). The samples were incubated again for 6 h, the medium was eliminated, and cells were then fixed with 5.0% (*w*/*v*) trichloroacetic acid. Several washed with cold ethanol were done, before the pellet was finally dissolved in 200 µL of 0.1 M NaOH containing 0.1% SDS. A Beckman LS3801 liquid scintillation counter was used to measure its radioactivity. Cytotoxicity was calculated in terms of IC_50_ values (namely, protein concentration needed to produce 50% protein synthesis inhibition), expressed as the percentage of the radioactivity incorporated in the assay. Three independent replicates per two assays were performed to average the IC_50_ values. When required, brefeldin A, that causes disassembly of Golgi-apparatus by altering vesicular trafficking [86], was added at 1 ng/mL together with the immunotoxins. A control, just with brefeldin A without the proteins, was performed to evaluate drug related toxicity.

## Figures and Tables

**Table 1:** Comparison of the results obtained for the furin variants vs. their originals counterparts, in relation to: level of colocalization with the Golgi apparatus and lysosomes; as well as the IC_50_ values obtained in the different cytotoxicity assays performed with just IMTXA33furαS or scFvA33furT1, or adding bafilomycin or brefeldin A, as described in Figure 8.

	**IMTXA33furαS**	IMTXA33αS *
Golgi colocalizationOverlap coefficient at 16 h (%)	18.2	46.3
IC_50_ (nM)	15	30–40
IC_50_ (nM) + Bafilomycin	15–20	30
IC_50_ (nM) + Brefefeldin A	5	>500
	**IMTXA33furT1**	IMTXA33T1 *
Lysosomes colocalizationOverlap coefficient at 16 h (%)	23.4	58.1
IC_50_ (nM)	300	>1 mM
IC_50_ (nM) + Bafilomycin	70–90	70–90
IC_50_ (nM) + Brefefeldin A	300	>1 mM

* Data from IMTXA33αS and scFVA33T1 used for comparison [39].

## Supplementary Materials

The following are available online at https://www.mdpi.com/2072-6651/11/10/593/s1, Figure S1: Structural characterization of Alexa-555 labelled furin-variant immunotoxins.
