# Supplementary material for: Inclusion of a Furin Cleavage Site Enhances Antitumor Efficacy against Colorectal Cancer Cells of Ribotoxin α-Sarcin- or RNase T1-Based Immunotoxins
Source: Toxins (Basel). 2019 Oct 12;11(10):593. doi: 10.3390/toxins11100593 (PMC6832446; doi:10.3390/toxins11100593)
Supplement: Supplementary file 1 [file toxins-11-00593-s001.pdf]

Supplementary Materials: Inclusion of a Furin Cleavage Site Enhances Antitumor Efficacy against Colorectal Cancer Cells of Ribotoxin α-Sarcin- or RNase T1-Based Immunotoxins

Javier Ruiz-de-la-Herrán, Jaime Tomé-Amat, Rodrigo Lázaro-Gorines, José G. Gavilanes and Javier Lacadena

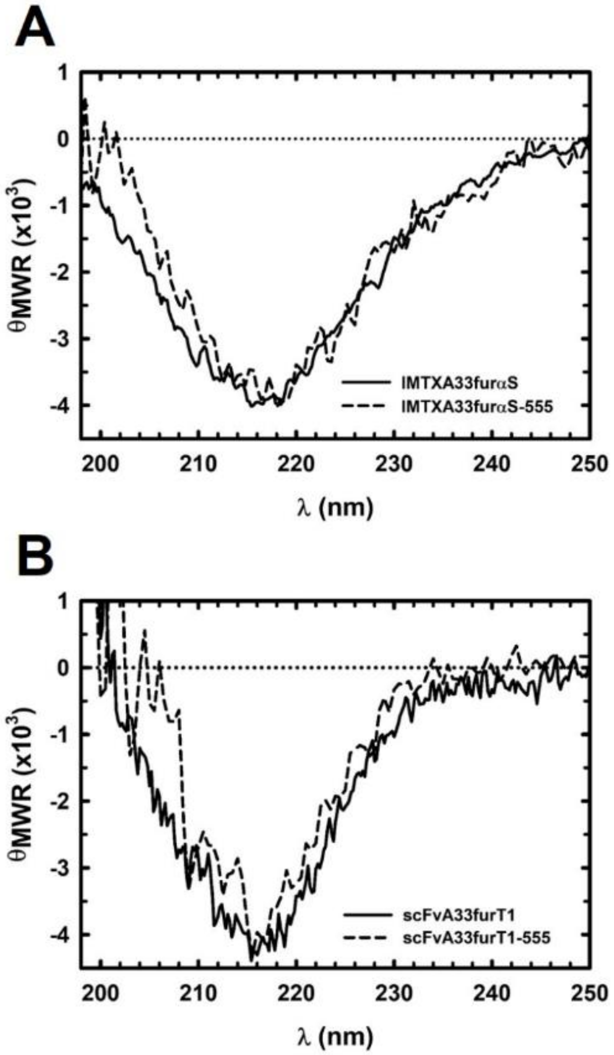

Figure S1. Structural characterization of Alexa-555 labelled furin-variant immunotoxins: Far-UV circular dichroism spectra (θ_{MRW}, mean residue weight ellipticities were expressed as degree x cm^2 x $dmol^{-1}$) of: (**A**) IMTXA33furαS (solid line) and IMTXA33furαS-555 (short dash line); (**B**) scFvA33furT1 and scFvA33furT1-555 (short dash line) and. Spectra were made with protein at 0.15 mg/ml in PBS.
